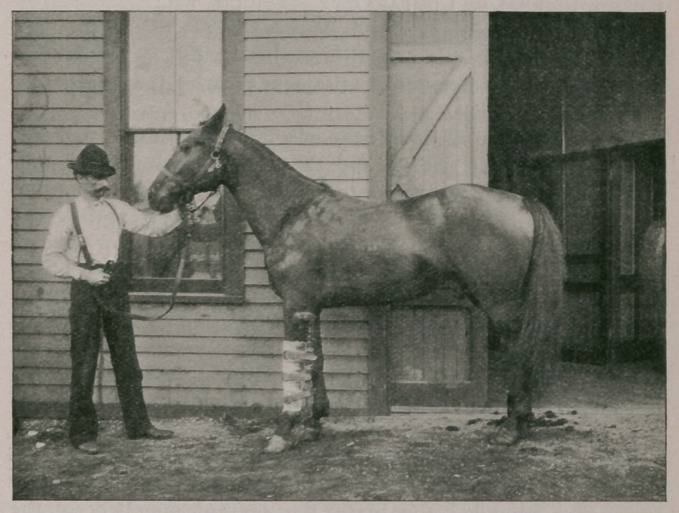# Fracture of the Trapezium

**Published:** 1896-04

**Authors:** John Wende

**Affiliations:** Buffalo, N. Y.


### FRACTURE OF THE TRAPEZIUM.

By JOHN WENDE, V.S.,
BUFFALO, N. Y.

AUGUST 10, 1895, I was called to attend a high-stepping carriage-horse owned by Mr. G. E. Drullard, of this city. Upon arriving at the stable the coachman informed me that the horse had run away that morning, and as he turned into the street, which was paved with asphalt and slippery at the time, he fell and was thrown against a stepping-stone on the opposite curb. Upon regaining his feet he was unable to pursue his frantic course on account of his inability to use his left foreleg. He was returned to his stable on three legs, where I saw him, holding his injured limb in a semiflexed position with toe resting on the floor, unable to bear any weight whatever upon it. Flexing the leg caused no inconvenience or pain, whereas extending it would cause him to rear upon his hind legs instantly.

Upon examination I found fracture of the trapezium. Applied cooling lotions to parts to prevent and keep down swelling, and had the patient removed to my infirmary per ambulance. Having no literature in my possession to which I could refer for a method of treatment in this case, I was forced to tax my own resources in every procedure. My first aim was to immobilize the injured member, which I did successfully with the assistance of Dr. F. A. Crandall, of this city. We first applied sheet cotton-batting, over which we rolled a dry bandage, upon this we placed two sole-leather splints that had been previously moulded to the limb. Over these we wound plaster-of-Paris bandages until they reached a thickness of about half an inch and extending from above the fetlock to the middle of the radius. A shoe had been previously applied to the foot with long heels welded together. The latter was bent sufficiently to elevate the posterior end about one inch from the floor, a hole was punched into the centre to receive the hook-end of an iron splint which extended up to the elbow-joint, where it was padded with felt. Three leather straps, two inches wide, running through loops on the splint, held the leg secure to the latter, as shown in cut.

The patient was placed in a sling, where he was kept until

October 8th, when splints were removed and he was walked home without any inconvenience or lameness, his exercise was gradually increased, and in three weeks he was able to do his work to the coach as well as before the accident, also without having lost any of the extremely high knee-action which he possessed before receiving the injury.

## Figures and Tables

**Figure f1:**